# Tryptanthrin Suppresses the Activation of the LPS-Treated BV2 Microglial Cell Line via Nrf2/HO-1 Antioxidant Signaling

**DOI:** 10.3389/fncel.2017.00018

**Published:** 2017-02-02

**Authors:** Young-Won Kwon, So Yeong Cheon, Sung Yun Park, Juhyun Song, Ju-Hee Lee

**Affiliations:** ^1^College of Korean Medicine, Dongguk UniversityGoyang, South Korea; ^2^Department of Anesthesiology and Pain Medicine, Yonsei University College of MedicineSeoul, South Korea; ^3^Department of Biomedical Sciences, Center for Creative Biomedical Scientists at Chonnam National UniversityGwangju, South Korea

**Keywords:** tryptanthrin, BV2 microglia cells, ischemic stroke, inflammation, nuclear factor erythroid 2-related factor 2 (Nrf2), heme oxygenase 1 (HO-1), nuclear factor-kappaB (NF-κB)

## Abstract

Microglia are the resident macrophages in the central nervous system (CNS) and play essential roles in neuronal homeostasis and neuroinflammatory pathologies. Recently, microglia have been shown to contribute decisively to neuropathologic processes after ischemic stroke. Furthermore, natural compounds have been reported to attenuate inflammation and pathologies associated with neuroinflammation. Tryptanthrin (indolo[2,1-b]quinazoline-6,12-dione) is a phytoalkaloid with known anti-inflammatory effects in cells. In present study, the authors confirmed middle cerebral artery occlusion (MCAO) injury triggers the activation of microglia in brain tissue, and investigated whether tryptanthrin influences the function of mouse murine BV2 microglia under LPS-induced inflammatory conditions *in vitro*. It was found tryptanthrin protected BV2 microglia cells against LPS-induced inflammation and inhibited the induction of M1 phenotype microglia under inflammatory conditions. In addition, tryptanthrin reduced the production of pro-inflammatory cytokines in BV2 microglia cells via nuclear factor erythroid 2-related factor 2 (Nrf2)/heme oxygenase 1 (HO-1) signaling and NF-κB signaling. The authors suggest that tryptanthrin might alleviate the progress of neuropathologies by controlling microglial functions under neuroinflammatory conditions.

## Introduction

Microglia are a type of mononuclear macrophage (Butovsky et al., 2012; Prinz and Priller, 2014) and are the main macrophages involved in inflammatory response in the central nervous system (CNS; Graeber and Streit, 2010), and reportedly, are important regulators of homeostasis and neuroinflammation (Cianciulli et al., 2016). Under stressful conditions, such as, in the presence of infection or inflammation, microglia are activated (Graeber and Streit, 2010), and secrete a variety of inflammatory mediators, such as, tumor necrosis factor-alpha (TNF-α), and interleukin (IL)-10, and mediators of inflammation, such as, reactive oxygen species (ROS), nitric oxide (NO) and prostaglandin E2 (PGE2; Streit et al., 2004; More et al., 2013). Accordingly, their activities are linked to the pathologies of numerous CNS diseases and conditions, such as, ischemic stroke (Mach et al., 1999; Hanisch and Kettenmann, 2007). When activated, microglia exhibit different phenotypes and perform different physiological roles (Boche et al., 2013; Cherry et al., 2014). The intracellular dynamics of microglia can be classified as M1 phenotype, which upregulates pro-inflammatory mediators (Gordon and Martinez, 2010), or as M2 phenotype, which upregulates anti-inflammatory mediators (Benarroch, 2013; Tang and Le, 2016). M1 microglia activation has been suggested to contribute to progressive tissue damage, leading to chronic neurodegeneration (Qin et al., 2013; Wang et al., 2013; Loane et al., 2014), whereas, M2 microglia activation has been reported to regulate wound healing and attenuate inflammation, and thus, contribute to tissue repair and regeneration (Cherry et al., 2014). In-ammation occurs throughout the progression of ischemic stroke and plays a crucial role in the neuropathological process (Picascia et al., 2015). After ischemic stroke, microglia retract finer processes (Kettenmann et al., 2011) and begin to produce inflammatory cytokines (Thored et al., 2009). Several studies have reported microglial activation in the penumbra area after middle cerebral artery occlusion (MCAO) injury (Rupalla et al., 1998; Ito et al., 2001; Perego et al., 2011). In recent years, to ameliorate pathology after stroke, many researchers have tried to regulate microglia function and changes in microglia phenotype during inflammatory conditions using various substances (Song et al., 2014; Zhang et al., 2015; Shu et al., 2016). Tryptanthrin (6,12-dihydro-6,12-dioxoindolo-(2,1-b)-quinazoline) is a natural alkaloid found in *P. tinctorium* (Honda et al., 1980; Scovill et al., 2002), and has been reported to have various pharmacological effects, such as, anti-inflammatory (Recio et al., 2006; Iwaki et al., 2011), antimicrobial (Honda et al., 1979) and anti-tumor effects (Kimoto et al., 2001; Yu et al., 2007, 2009; Liao et al., 2013). Tryptanthrin has also been reported to suppress NO and prostaglandin E synthesis in macrophages exposed to oxidative stress (Ishihara et al., 2000). In present study, we investigated whether tryptanthrin affects the phenotype and function of BV2 microglial cell line, which has been used as a common microglia cell line, under inflammatory conditions. Initially, we confirmed MCAO injury triggers the activation of microglia in brain tissue, and then sought to determine whether tryptanthrin influences the function of microglia under LPS-induced inflammatory conditions *in vitro*.

## Materials and Methods

### MCAO Mouse Model

To study the effect of stroke on brain tissue, we used MCAO mouse model. Male C57BL/6 mice (Orient, GyeongGi-Do, Korea; 8 weeks old) were used in this study. Mice were anesthetized with 5% isoflurane and then maintained with 2% isoflurane in 30% oxygen and 70% nitrous oxide by a face mask onto homeothermic blanket for maintaining rectal temperature 37 ± 0.5°C. Mice were subjected to transient focal cerebral ischemia by blocking middle cerebral artery with a 6–0 nylon suture. After 60 min of MCAO, blood flow was restored by withdrawing the suture, and cerebral blood flow was monitored using a Doppler flow meter (Transonic Systems, Inc., Ithaca, NY, USA). After reperfusion for 8 h, Zoletil mixture (30 mg/kg; Virvac Laboratories, Carros, France) was injected intraperitoneally. Mice were cardiac-perfused with saline and brains were extracted. All animal care and experimental procedures were conducted in accordance with the guidance for animal experiments established by the Yonsei University. The animals were housed in an air-conditioned room at 25°C with a 12 h dark/light cycle. All animals received human care with unlimited access to chow and water.

### Cell Culture and Drug Treatment

To check the tryptanthrin’s effect on microglia, we used mouse murine BV2 microglial cells. BV2 microglia cells were cultured in Dulbecco Modified Eagle Medium (DMEM; Gibco, Grand Island, NY, USA) supplemented with 100 μg/ml penicillin-streptomycin (Gibco, Grand Island, NY, USA) and 10% fetal bovine serum (Gibco, Grand Island, NY, USA) at 37°C in 5% CO_2_ incubator. For all experiments, cells were grown to 80%–90% confluency and then subjected to no more than 20 cell passages. Tryptanthrin was purchased from Sigma-Aldrich (St. Louis, MO, USA). Protoporphyrin IX (Snpp; as an inhibitor of heme oxygenase 1, HO-1) was purchased from Porphyrin Products (Logan, UT, USA). BV2 cells were serum-starved for 4 h, and then treated with tryptanthrin for 1 h prior to the addition of LPS (1 μg/ml), and the cells were further incubated for the next 8 h. On the other hand, other BV2 cells were first treated with 50 nM SnPP for 30 min, tryptanthrin (20 μM) for 1 h and then exposed to LPS (1 μg/ml) for 8 h.

### 3-(4,5-dimethylthiazol-2-yl)-2,5-diphenyltetrazolium bromide (MTT) Assay

BV2 microglia cells were plated at 1 × 10^4^ cells/well in 96-well plates. After incubation for 1 day, cells were treated with either dimethyl sulfoxide (DMSO) as the control or with various concentration (0.1–50 μM) of tryptanthrin. The final concentration of DMSO in the medium was 0.1% and DMSO had no effect on cell viability. After exposure to tryptanthrin for 24 h, viable cells were stained with 3-(4,5-dimethylthiazol-2-yl)-2,5-diphenyltetrazolium bromide (MTT; Sigma-Aldrich, St. Louis, MO, USA) solution (2 mg/ml in PBS). After 3 h of incubation, medium was removed, and 100 μl of DMSO was added to solubilize the formazan crystals. The absorbance was measured at 570 nm using a multimode microplate reader (Tecan, Research Triangle Park, NC, USA). The analysis was conducted in triplicate. Cell viabilities were defined relative to control cells (considered to be 100%).

### Determination of Nitrite

BV2 microglia cells were plated onto 96-well plates, pre-treated with different concentrations of tryptanthrin (0.1–20 μM) for 1 h, and then stimulated with 1 μg/ml of LPS. Supernatants were collected and assayed for NO production by adding Griess reagent (100 μl/well) and incubating for 15 min at room temperature. Supernatant absorbance was measured at 540 nm using a microplate reader (Versamax, Molecular Devices; Cendan et al., 1996; Mazzio et al., 2016).

### Cytokine Assay (ELISA Assay)

BV2 microglia cells were plated in 6-well plates (5 × 10^5^ cells/ml) and incubated with tryptanthrin in the presence of LPS for 24 h. Cell-free supernatants were then collected, and IL-6 and TNF-α levels were measured using a BD OptEIA^TM^ Mouse IL-6 ELISA Kit (BD Biosciences, San Diego, CA, USA, Cat No 550950) or a Cymax Mouse TNF-α ELISA kit (AbFrontier, Seoul, Korea, Cat No LF-EK0275), respectively. Absorbances at 450 nm were measured using an ELISA microplate reader.

### Western Blot Analysis

BV2 microglia cells were washed rapidly with ice-cold PBS, scraped, and collected. BV2 cell pellets were lysed with ice-cold RIPA buffer (Thermo Scientific, Rockford, IL, USA) containing protease/phosphatase inhibitor cocktail (GenDEPOT, Barker, TX, USA). The lysates were centrifuged at 13,000 rpm for 30 min at 4°C to produce whole-cell extracts, and protein concentrations were then assessed using a bicinchoninic acid (BCA) assay. Proteins (30 μg) were separated on 10% SDS-polyacrylamide gel and transferred onto polyvinylidene difluoride (PVDF) membranes (Millipore, Bedford, MA, USA). After blocking with skim milk prepared in TBS/Tween (20 mM Tris (pH 7.2), 150 mM NaCl, 0.1% Tween 20) for 1 h at room temperature, immunoblots were incubated for 18 h at 4°C with primary antibodies that specifically detected cyclooxygenase-2 (COX-2; M19; sc-1747-R) Rabbit polyclonal (1:1000; Santa Cruz Biotechnology, Santa Cruz, CA, USA; Almada et al., 2016), inducible NO synthase (iNOS; AB5382) Rabbit polyclonal (1:1000; EMD Millipore, Temecula, CA, USA; Stansley et al., 2012), HO-1 (A-3; sc-136960) mouse monoclonal (1:1000, Santa Cruz Biotechnology; Almada et al., 2016), or β-actin (C4; sc-47778) mouse monoclonal (1:2000; Santa Cruz Biotechnology; Wang et al., 2016). Brain tissues were lysed with ice-cold RIPA buffer (Thermo Scientific, Rockford, IL, USA) containing protease/phosphatase inhibitor cocktail (GenDEPOT, Barker, TX, USA), and supernatants were centrifuged at 13,000 rpm for 1 h 30 min at 4°C. Proteins (30 μg) were separated on 10% SDS gel and transferred to PVDF membranes. After blocking for 1 h at room temperature, immunoblots were incubated for 12 h at 4°C with primary antibodies that specifically detected iNOS (AB5382) Rabbit polyclonal (1:1000; EMD Millipore, Temecula, CA, USA; Stansley et al., 2012), IkBα (L35A5; #4814) mouse monoclonal (1:1000, Cell Signaling, Danvers, MA, USA; Horvath et al., 2008), or p-NF-κB (Ser276; #3037) Rabbit polyclonal (1:500, Cell Signaling, Danvers, MA, USA; Henn et al., 2009). Blots were then incubated with each secondary antibody (Cell Signaling, Danvers, MA, USA) for 1 h at room temperature. Blots were visualized by ECL (Amersham Biosciences, Piscataway, NJ, USA). Equal loading of proteins was verified by actin immunoblottings. At least three separate experiments were performed to confirm changes. Band intensities were quantified using ImageJ 1.42 software (NIH Bethesda, MD, USA). Band densitometry was analyzed by arbitrarily drawing a rectangular box around the selected protein bands of interest. The equal sized rectangular box was used to measure all bands of interest and background-subtracted density of each peak of interest is quantified. All densitometry data are normalized to the respective band intensity of the β-actin loading controls. Nuclear and cytosolic fractions were prepared using NE-PER^TM^ Nuclear and Cytoplasmic Extraction Reagents (Thermo Scientific, Rockford, IL, USA) according to the manufacturer’s instructions. Briefly, cells were lysed in ice-cold CER-I buffer supplemented with protease inhibitors and incubated on ice for 10 min. After addition of ice-cold CER-II, samples were centrifuged and the supernatant solution (cytoplasmic fraction) was collected. Pellets containing nuclei were washed three times in PBS to remove contamination of cytosolic proteins and nuclear proteins were extracted in NER buffer supplemented with protease inhibitors.

### Immunocytochemistry

BV2 microglial cells onto the coated glass were fixed with 4% paraformaldehyde (PFA) for 30 min and washed three times with PBS. BV2 cells were permeabilized with 0.3% Triton X-100 in PBS for 15 min, and blocked with 5% bovine serum albumin (BSA) for 1 h. And then, samples were incubated with primary antibodies overnight at 4°C. The following primary antibodies were used: anti-mouse CD68 (1:500, Santa Cruz Biotechnology, Santa Cruz, CA, USA; Blasi et al., 1990), anti-goat p-NF-κB (1:500, Cell Signaling, Danvers, MA, USA; Henn et al., 2009), and anti-mouse nuclear factor erythroid 2-related factor 2 (Nrf2; 1:500, Santa Cruz Biotechnology, Santa Cruz, CA, USA; Zhang et al., 2016). After incubation, cells were washed three times for 3 min with PBS, incubated with each secondary antibody for 1 h at room temperature, and counterstained with 1 μg/ml 4′,6-diamidino-2-phenylindole (DAPI, 1:200, Invitrogen, Carlsbad, CA, USA; Smolewski et al., 2001) for 15 min at room temperature. Cells were imaged under a Zeiss LSM 710 confocal microscope (Carl Zeiss, Thornwood, NY, USA; Cheon et al., 2016). The images were acquired with objective lens of 40× as composites. Immunofluorescence was observed at Excitation/Emission (nm; 358/463) for DAPI, (495/517) for FITC, and (551/573) for Rhodamine by smart setup system in ZEN microscope and Imaging software (Zeiss LSM 710, Carl Zeiss, Oberkochen, Germany). Nrf2 immunoreactivity was measured using ImageJ (ImageJ, Bethesda, MD, USA) by analyzing the mean intensity of images obtained from single confocal planes average intensity and applying a manual selected threshold above the background intensity. Mean intensity of p-NF-κB immunoreactivity was analyzed in Z-stack average intesity by using Zen 2010 software from Carl Zeiss. Nuclear p-NF-κB was identified by its colocalization with DAPI staining while no colocalizing p-NF-kB was considered cytosolic. Immunofluorescent intensity for p-NF-κB indicated Mean intensity of three slice of projection.

### Immunohistochemistry

Twenty-micrometer-thick frozen brain sections were cut onto clean glass slides (Thermo Scientific, Waltham, MA, USA), air-dried, and subsequently post-fixed in acetone for 15 min at −20°C. The slides were first washed in PBS and dipped into −20°C ethyl alcohol for 20 min, incubated with 0.3% Trion X-100 which diluted in PBS. After blocking nonspecific labeling, sections were incubated in 5% BSA (Sigma-Aldrich, St. Louis, MO, USA) diluted in PBS for 30 min, and then primary and secondary antibodies were added. Primary antibodies for CD68 (1:50, Santa Cruz Biotechnology, Santa Cruz, CA, USA; Blasi et al., 1990) were applied to samples overnight at 4°C, and incubated for 2 h in the dark at room temperature with FITC-conjugated anti-mouse Ig secondary antibody (1:100, Invitrogen, Carlsbad, CA, USA). The sections again washed three times in PBS. Sections were counterstained with 1 μg/ml DAPI (Invitrogen, Carlsbad, CA, USA; Smolewski et al., 2001) and examined under a confocal microscope (Zeiss LSM 710, Carl Zeiss, Oberkochen, Germany). The images were acquired with objective lens of 40× as composites. Immunofluorescence was observed at Excitation/Emission (nm; 358/463) for DAPI and (495/517) for FITC by smart setup system in ZEN microscope and Imaging software (Carl Zeiss). Three optical fields in a 150 × 150 μm of the striatum and cortex (Bregma 0.62 mm) were randomly chosen under a 40× objective from each sample for quantification (**Supplementary Figure** S1). The percentage of positive cells for CD68 was measured by ImageJ (ImageJ, Bethesda, MD, USA) program. The minimal number of cells counted per mouse was 100. The percentage of CD68 positive cells was calculated dividing the total number of CD68 positive cells by the total number of DAPI stained cells observed in the same field.

### Statistical Analysis

Results are presented as mean ± standard deviations (SDs). Statistical significances were determined using one-way analysis of variance (ANOVA) followed by Turkey’s multiple comparison and Student’s *t*-test. All experiments were independently conducted at least three times. Data were analyzed with Prism 5.0 software (GraphPad Software, Inc., San Diego, CA, USA), and statistical significance was accepted for *p* values < 0.05.

## Results

### MCAO Injury Triggered the Activation of Microglia in Brain

**Figure 1:**
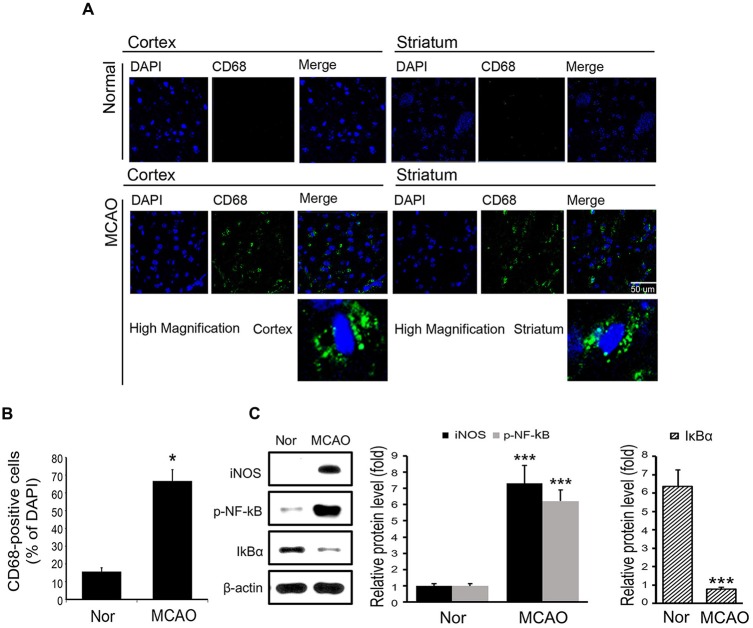
**Expression levels of CD68, inducible NO synthase (iNOS), p-NF-κB and IκBα in mouse brain tissues. (A)** Levels of CD68 (a marked of activated microglia) in brain tissues were assessed immunohistochemically. Images show CD68-positive cells (green) in the cortex of middle cerebral artery occlusion (MCAO) mice. CD68 expression was considerably higher in cortex in the 8 h MCAO mouse brain than in the control mouse brain. In addition, CD68-positive cells were observed in ischemic striatum. **(B)** A plot of CD68-positive cell percentages. Results are expressed as means ± SDs. Each experiment conducted 3 mice per conditions. Significant vs. normal, *p < 0.05 (paired *T*-Test with MCAO mice. **(C)** The protein levels of iNOS, p-NF-κB and IκBα in brain. Data are expressed as mean ± SDs, and each experiment conducted three repeats per conditions. Significant vs. normal, ***p < 0.001. Scale bar = 50 μm, CD68: green, 4′,6-diamidino-2-phenylindole (DAPI): blue, Nor: normal control group, MCAO: reperfusion 8 h after MCAO injury.

To examine microglia activation in mouse brain tissues, we checked the expression of CD68 (Song and Lee, 2015) by immunostaining (Figure 1A). Figure 1B showed the positive cells percentage of CD68 (Figure 1B). These data showed that MCAO resulted in microglia activation in brain cortex and striatum (Figures 1A,B). **Supplementary Figure** S1 showed the position of brain for immunostaining (**Supplementary Figure** S1).

### MCAO Injury Promoted Inflammatory Signaling in Brain

To examine inflammatory signaling, we measured the protein levels of iNOS and of phosphorylated NF-κB in MCAO mouse brain tissues (Figure 1C). It was found the protein levels of iNOS and phosphorylated NF-κB were substantially increased in MCAO mouse brain tissue as compared with normal brain tissue (Figure 1C). In addition, the protein levels of IκBα, which binds with NF-κB in cytosol to inhibit the activation of NF-κB (Forman et al., 2016), were significantly lower in MCAO mouse brain tissue than in normal brain tissue (Figure 1C). These observations show that MCAO injury activates iNOS and NF-κB signaling.

### Tryptanthrin Reduced the Production of Nitric Oxide (NO) and Attenuated Inflammatory Signaling in BV2 Microglia Under Inflammatory Conditions

**Figure 2:**
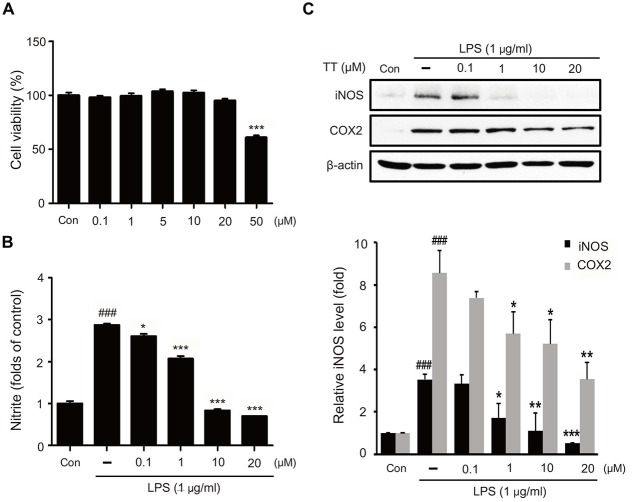
**Effects of tryptanthrin on cell viability, nitric oxide (NO) production, and on the expressions of iNOS and COX-2. (A)** Cell viability of tryptanthrin treated microglia. BV2 microglia cells were treated with tryptanthrin at 0.1, 1, 10, 20, or 50 μM for 24 h. At concentrations below 50 μM tryptanthrin treatment resulted in cell viabilities of >90%, and at 50 μM cell viability was ~60%. Results are expressed as means ± SDs. Significant vs. control, ***p < 0.001 (ANOVA analysis). **(B)** Nitrite production by LPS treated microglia was measured using Griess reagent. Significant vs. control, ^###^*p* < 0.001; significant vs. LPS, *p < 0.05, ***p < 0.001 (ANOVA analysis). **(C)** Western blotting showed that the protein levels of iNOS and COX-2 were considerably higher in LPS treated BV2 microglia than in non-treated controls. Furthermore, tryptanthrin pre-treatment reduced LPS-induced increases in iNOS and COX-2 protein levels. β-actin was used as an internal control. Each experiment conducted three repeats per conditions. Significant vs. control, ^###^*p* < 0.001; significant vs. LPS, *p < 0.05, **p < 0.01, ***p < 0.001 (ANOVA analysis). TT, tryptanthrin.

To assess the cellular toxicity of tryptanthrin, we measured the cell viability of BV2 microglia cells treated with tryptanthrin for 24 h using a MTT assay (Figure 2A). Our results showed tryptanthrin did not affect cell viability at concentrations of 0 μM to 20 μM (Figure 2A). In addition, we found that NO production was increased in BV2 microglia cells after 8 h of LPS treatment (Figure 2B). To assess the activations of iNOS and COX-2 inflammatory signals in LPS treated BV2 microglia cells, we conducted western blotting analysis (Figure 2C). Our results showed that the protein levels of iNOS and COX-2 were markedly increased in LPS treated BV2 microglia cells, and that tryptanthrin pretreatment inhibited these increases (Figure 2C). At a concentration of 20 μM, tryptanthrin induced marked decreases in LPS-induced increases in iNOS and COX-2 protein levels (Figure 2C).

### Tryptanthrin Inhibited the Induction of M1 Phenotype Microglia Under Inflammatory Conditions

**Figure 3:**
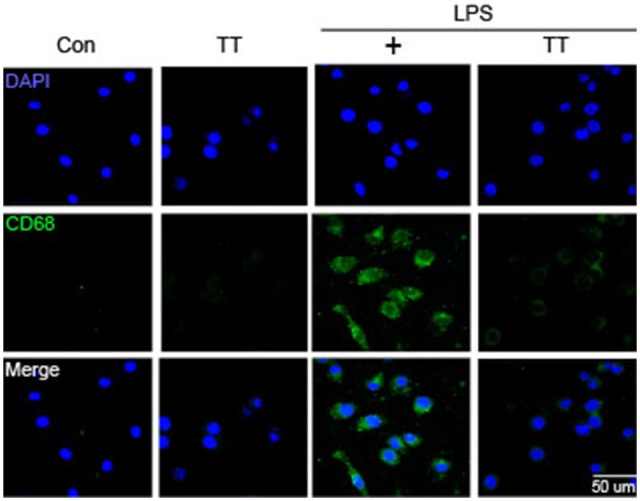
**CD68 expression in LPS-treated BV2 microglia.** Immunocytochemistry was used to investigate the expression of CD68 (a marker of microglia activation). CD68 expression was higher in LPS treated BV2 microglia than in non-treated controls, and tryptanthrin pretreatment attenuated this LPS-induced expression. Scale bar: 50 μm, 4′,6-diamidino-2-phenylindole (DAPI): blue, CD68: green, Con, normal control group; LPS, LPS (1 μg/ml) treatment group; TT, tryptanthrin.

**Figure 4:**
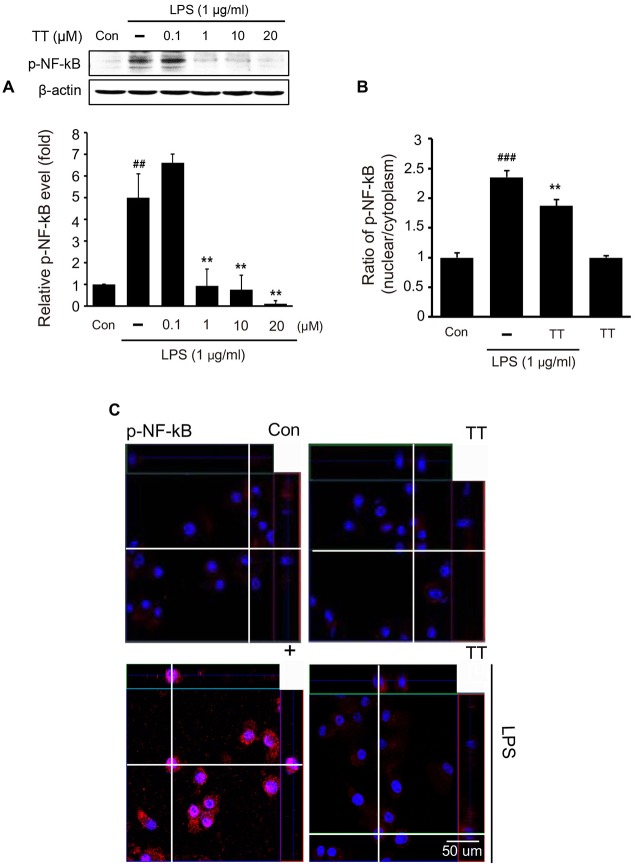
**NF-κB activation in tryptanthrin pretreated BV2 microglia. (A)** Western blotting showed the protein level of p-NF-κB p65 was increased by treating naïve cells with LPS, but that tryptanthrin pretreatment inhibited this LPS-induced increase. β-actin was used as a loading control. **(B)** Ratio of p-NF-κB (nucleus/cytoplasm) was measured by Zen program (Zen program, Oberkochen, Germany). This showed the translocation ratio of NF-κB into nucleus. **(C)** NF-κB activation was assessed immunocytochemically. The image shows the nuclear translocation of NF-κB p65 in LPS-treated BV2 microglia as compared with non-treated controls. Tryptanthrin (20 μM) pretreatment inhibited the LPS-induced nuclear translocation of NF-κB p65. Results are expressed as means ± SDs. Each experiment conducted four repeats per conditions. Significant vs. control, ^##^*p* < 0.05, ^###^*p* < 0.001; significant vs. LPS, **p < 0.01 (ANOVA analysis). Con, non-treated control; TT, tryptanthrin; 4′,6-diamidino-2-phenylindole (DAPI): blue, p-NF-κB: red.

**Figure 5:**
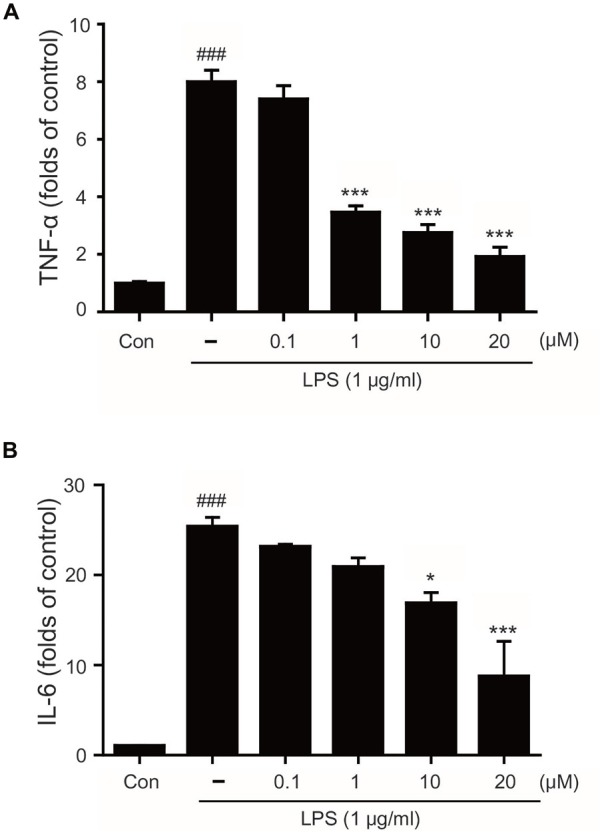
**Effects of tryptanthrin on LPS-induced cytokine production in BV2 microglia. (A)** LPS-induced tumor necrosis factor-alpha (TNF)-α production and **(B)** LPS-induced interleukin (IL)-6 production were also reduced by pretreating cells with tryptanthrin. Each experiment conducted four repeats per conditions. Significant vs. control, ^###^*p* < 0.001; significant vs. LPS, *p < 0.05, ***p < 0.001 (ANOVA analysis). Con, non-treated control.

To determine whether tryptanthrin promotes phenotype change of microglia under inflammatory conditions, we observed the expression of mannose receptor CD68 (a marker of activated microglia) by immunostaining (Figure 3). Our data indicated that LPS treatment increased the expression of CD68 in BV2 microglia cells, and that tryptanthrin pretreatment suppressed this increase (Figure 3). Our western blotting data also showed that tryptanthrin attenuated the LPS-induced phosphorylation of NF-κB p65 (Figure 4A). The phosphorylation of NF-κB was reduced by tryptanthrin in LPS treated BV2 microglia (Figure 4A). To confirm that tryptanthrin controls the activation of NF-kB signaling in BV2 microglia, we checked the phosphorylation and nuclear translocation of NF-κB p65 in tryptanthrin pretreated BV2 microglia under LPS induced inflammatory condition by immunostaining (Figures 4B,C). It was found tryptanthrin suppressed the LPS-induced activation of NF-κB (Figures 4A–C). To determine whether tryptanthrin is involved in the secretion of cytokines by LPS treated BV2 microglia, we assessed cytokine production by ELISA (Figure 5). The productions of TNF-α and IL-6 were found to be markedly reduced by tryptanthrin in LPS treated BV2 microglia (Figure 5). Consequently, our results indicate that tryptanthrin suppresses production of pro-inflammatory cytokines in BV2 microglia under inflammatory conditions (Figure 5).

### Tryptanthrin Attenuated the Production of NO and Cytokines through Nrf2/HO-1 Signaling in LPS Treated BV2 Microglia

**Figure 6:**
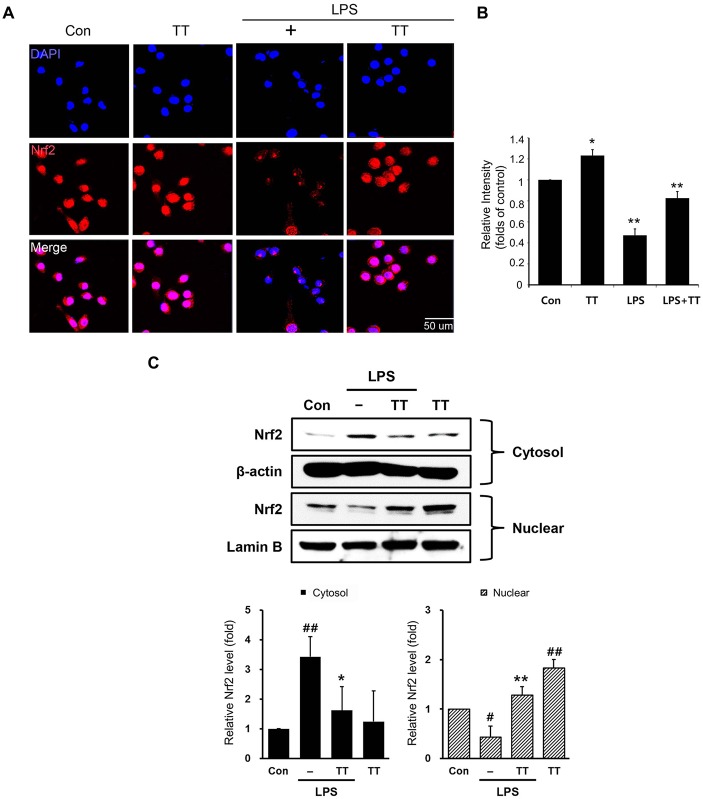
**Nuclear factor erythroid 2-related factor 2 (Nrf2)/heme oxygenase 1 (HO-1) signaling pathway activation in tryptanthrin pre-treated BV2 microglia. (A)** Immunostaining data showing the expression of Nrf2 was greater in tryptanthrin pretreated BV2 microglia than in BV2 microglia treated with LPS alone. **(B)** The graph of Nrf2 immunostaining relative intensity in BV2 microglia. **(C)** Western blotting data showed that the translocation of Nrf2 from cytosol into nucleus using nucleus fraction. Each experiment conducted four repeats per conditions. Results are expressed as means ± SDs. Results are expressed as means ± SDs. Significant vs. control, ^#^*p* < 0.05, ^##^*p* < 0.01; significant vs. LPS, *p < 0.05, **p < 0.01 (ANOVA analysis). Scale bar: 50 μm, 4′,6-diamidino-2-phenylindole (DAPI): blue, Nrf2: red. Con, non-treated control; LPS, LPS (1 μg/ml) treated cells; TT, tryptanthrin.

**Figure 7:**
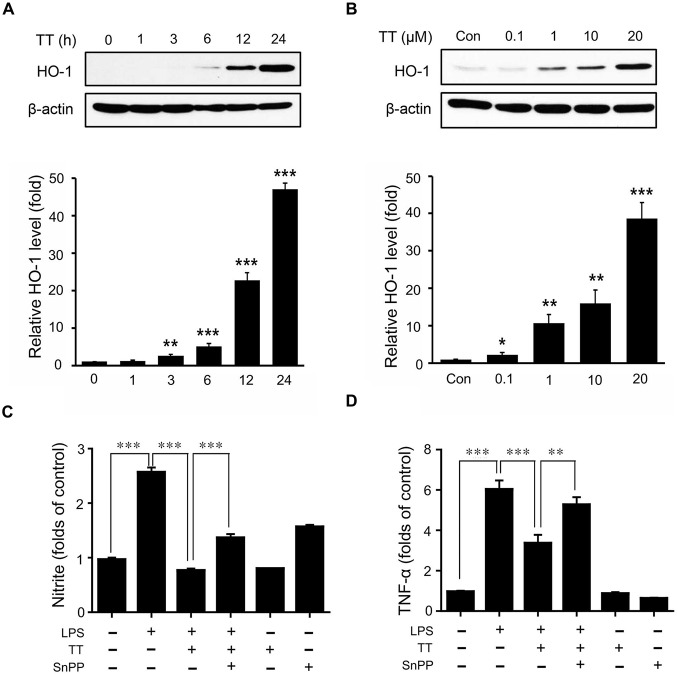
**Effects of tryptanthrin on HO-1 expression in BV2 microglia.** **(A)** BV2 microglial cells were incubated with 20 μM tryptanthrin for 0–24 h. **(B)** BV2 microglia cells were treated for 8 h with 0.1–20 μM concentrations of tryptanthrin. HO-1 expression was assessed by western blotting using a specific antibody. Tryptanthrin time- and dose-dependently induced HO-1 expression. β-actin was used as an internal loading control. To examine the inhibitory effects of tryptanthrin pretreatment and of co-pretreatments with tryptanthrin and SnPP (a HO-1 inhibitor) on the LPS-induced productions of nitrite **(C)** and TNF-α **(D)**, BV2 microglia cells were pre-treated with tryptanthrin (20 μM) for 1 h in the presence or absence of SnPP (50 nM, 30 min), and then stimulated with LPS (1 μg/ml) for 8 h. Each experiment conducted four repeats per conditions. Levels of nitrite and TNF-α are presented as means ± SDs. *p < 0.05, **p < 0.01, ***p < 0.001 (ANOVA analysis). Con, non-treated control; TT, tryptanthrin; LPS, LPS 1 μg/ml treatment; SnPP, 50 nM SnPP treatment.

To examine the activation of Nrf2/HO-1 signaling in LPS treated microglia, we used immunocytochemistry (Figures 6A,B) and western blotting (Figures 6C, 7). The expression of Nrf2 in LPS treated BV2 microglia was lower than in normal BV2 microglia, whereas tryptanthrin tends to induce the activation of Nrf2 in inflammatory condition (Figures 6A,B). Figure 6C showed treatment with tryptanthrin resulted in the nuclear translocation of Nrf2 in the absence or presence of LPS (Figure 6C). To confirm whether tryptanthrin regulates HO-1 induction in BV2 microglia under inflammatory conditions, we checked the induction of HO-1 in BV2 microglia by western blotting (Figures 7A,B). The induction of HO-1 was evidently promoted by tryptanthrin (Figure 7A). We also observed that the inhibition of HO-1 induction by SnPP leads to the increase of nitrite (Figure 7C) and TNF-α (Figure 7D) in tryptanthrin pretreated BV2 microglia under LPS-induced inflammatory condition.

## Discussion

The present study was undertaken to confirm MCAO injury triggers the activation of microglia in brain tissue, and to investigate whether tryptanthrin influences the function of BV2 microglia under LPS-induced inflammatory conditions *in vitro*. The study confirms MCAO induces the activation of microglia in brain tissue, and shows tryptanthrin reduces neuroinflammatory response by controlling microglial function. Taken together, tryptanthrin appears to ameliorate the pro-inflammatory effects of BV2 microglia and to have therapeutic potential for the prevention of brain tissue damage in the presence of neuroinflammatory disease. Ischemic stroke causes damage by triggering severe inflammation in brain (Roger et al., 2012). In ischemic stroke, microglia activate and promote the productions of inflammatory cytokines and the activation of inflammatory signaling (Lakhan et al., 2009). In present study, we confirmed the activation of microglia in our MCAO model of ischemic stroke (Meng et al., 2016). In addition, we observed LPS induce inflammation leads to the induction of M1 phenotype microglia in brain based on the increased expression of CD68 mannose receptor and iNOS in brain (Lisi et al., 2014; Meng et al., 2016). M1 phenotype microglia are associated with the activation of inflammatory signaling, such as, by NF-κB and iNOS, which promotes the productions of pro-inflammatory cytokines and could lead to severe neuropathology and pain (Popiolek-Barczyk et al., 2015; Meng et al., 2016). Our *in vivo* data suggest MCAO injury as an inflammatory injury leads to M1 induction in brain tissues and trigger the activations of NF-κB and iNOS inflammatory signaling. Tryptanthrin (a natural alkaloid) has strong anti-inflammatory effects and has been shown to protect cells against stressful conditions (Recio et al., 2006; Iwaki et al., 2011; Pathania et al., 2014; Popov et al., 2015). We employed BV2 microglia cell line to evaluate the effects of tryptanthrin on microglia. BV2 microglia cell line derived from *v-raf*/*v-myc*-immortalized murine neonatal microglia (Blasi et al., 1990) has been frequently used substitute for primary microglia (Stansley et al., 2012). There are concerns about suitability of BV2 cells as a sufficient substitute for primary microglia. However, recent studies have found that their responses to LPS are similar to primary microglia on the gene profiling and functional capacities in NO and cytokine secretion (Horvath et al., 2008; Henn et al., 2009). Furthermore, they have suggested that BV2 cells are suitable to primary microglia and animal experiments due to the price of the assay and the speed of data generation (Henn et al., 2009). Our *in vitro* data confirm tryptanthrin influences the polarization of BV2 microglia and could reduce the induction of the M1 phenotype under LPS induced inflammatory conditions. Furthermore, we found tryptanthrin attenuated the secretion of NO and the activations of iNOS and COX-2. Several studies have demonstrated NO plays a crucial role in promoting inflammatory response (Wang et al., 2007), and that reactive microglia express iNOS leading to energy depletion and cell death after ischemic stroke (Samdani et al., 1997; Saha and Pahan, 2006). We observed tryptanthrin treatment under inflammatory condition decreased pro-inflammatory cytokine levels, including those of TNF-α and IL-6. Given that pro-inflammatory cytokines promote COX-2 and iNOS transcription (Das and Basu, 2008; Perry et al., 2010), we believe tryptanthrin may inhibit the activations of COX-2 and iNOS by reducing the secretions of pro-inflammatory cytokines. Furthermore, our results indicate that tryptanthrin blocks NF-κB transcriptional activity in BV2 microglia under inflammation conditions. NF-κB is an important transcription factor in the regulation of inflammatory response, and its activation causes cellular death (Camandola and Mattson, 2007). In addition, NF-κB has been known to suppress the expressions of iNOS and COX-2 and to activate pro-inflammatory cytokine secretion (Shin et al., 2002; Atreya et al., 2008; Nam et al., 2010). Previous studies have demonstrated that inhibition of NF-κB in mouse brain reduce brain infarct volumes after ischemic injury (Nurmi et al., 2004; Qin et al., 2007). Although we did not find tryptanthrin reduced brain infarct volumes in our MCAO model, we assume that tryptanthrin may have the potential to attenuate neuropathology of ischemic stroke as an inflammatory neurodisease by inhibiting NF-κB signaling in microglia. HO enzymes are known to be crucial components of the cellular antioxidant system and to be involved in various diseases, including ischemic stroke (Panahian et al., 1999), and HO-1 induction by activated Nrf2 (Qiang et al., 2004) protects cells against oxidative stress (de Vries et al., 2008). In addition, HO-1 in microglia has been reported to have an anti- inflammatory effect (Min et al., 2008), for example, it inhibits pro-inflammatory cytokine secretion by activated microglia (Petrache et al., 2000). Furthermore, the expressional up-regulation of HO-1 was found to reduce the expressions of pro-inflammatory cytokines and suppress COX-2 and iNOS levels (Suh et al., 2006). Interestingly, it has also been demonstrated that tryptanthrin upregulates HO-1 signaling in hepatocytes (Moon et al., 2014). In present study, tryptanthrin attenuated the production of pro-inflammatory cytokines and nitrite in BV2 microglia through Nrf2/HO-1 signaling. Although we did not examine behavioral improvements or attempt to quantify brain damage reduction by tryptanthrin in our MCAO model, the study provides evidence tryptanthrin might control the function of microglia through Nrf2/HO-1 and NF-κB signaling, which suggests tryptanthrin could alleviate neuropathology of neuroinflammatory disease such as stroke. To confirm these findings, various preclinical studies need to be performed to investigate the toxicological as well as the potential preventive or even therapeutic effects of tryptanthrin.

## Author Contributions

Y-WK, SYC and SYP conducted the experiments. Y-WK and SYC contributed to the writing of the first draft of the manuscript. JS and J-HL designed the study and wrote the manuscript, and provided overall supervision for the project.

## Conflict of Interest Statement

The authors declare that the research was conducted in the absence of any commercial or financial relationships that could be construed as a potential conflict of interest.

## Supplementary Material

Figure S1 **The atlas of brain position.** For this study, we measured the expressions of CD68 in bregma 0.62 mm of MCAO mouse. This image is from brain atlas.
