# Supplementary figures and images for: Tryptanthrin Suppresses the Activation of the LPS-Treated BV2 Microglial Cell Line via Nrf2/HO-1 Antioxidant Signaling
Source: Front Cell Neurosci. 2017 Feb 2;11:18. doi: 10.3389/fncel.2017.00018 (PMC5288339; doi:10.3389/fncel.2017.00018)

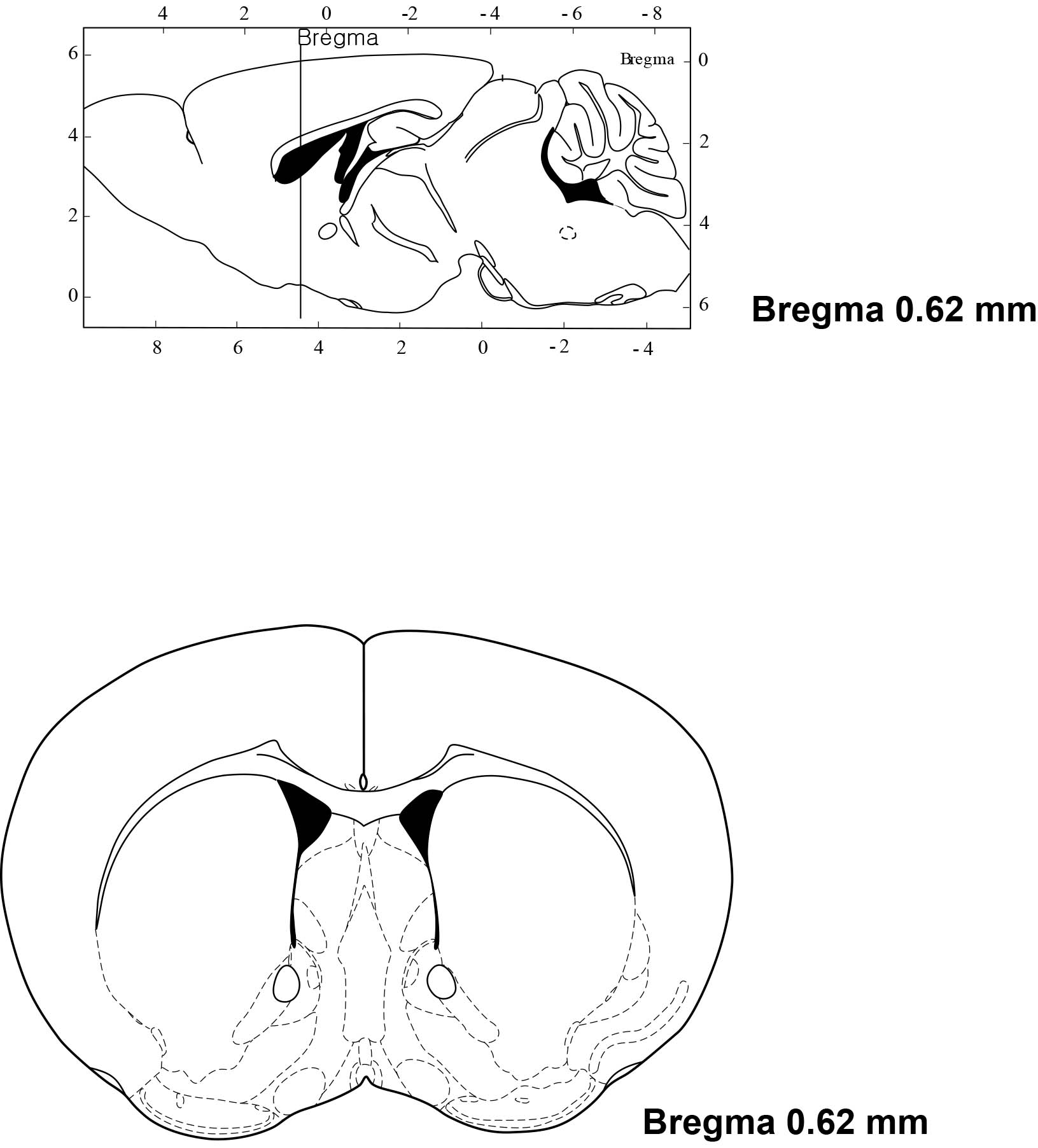

Supplement: Figure S1 — The atlas of brain position. For this study, we measured the expressions of CD68 in bregma 0.62 mm of MCAO mouse. This image is from brain atlas. [file Image_1.jpeg]
